# Impact of Antimicrobial Stewardship on Reducing Antimicrobial Resistance

**DOI:** 10.7759/cureus.49935

**Published:** 2023-12-04

**Authors:** Sagar N Khadse, Sarita Ugemuge, Charu Singh

**Affiliations:** 1 Medical Education, Datta Meghe Medical College, Datta Meghe Institute of Higher Education and Research, Nagpur, IND; 2 Microbiology, Datta Meghe Medical College, Datta Meghe Institute of Higher Education and Research, Nagpur, IND

**Keywords:** infection control, prescription guidelines, diagnostic advancements, aware classification, antibiotic de-escalation, hospital formulary management, antimicrobial stewardship program, antimicrobial resistance

## Abstract

Antimicrobial resistance has become a serious global issue, posing a significant threat to public health and healthcare professionals. Since the advent of penicillin, many antibiotics have lost their effectiveness in combating microbes simply due to inappropriate, irrational, unnecessary, and unrestricted use. The ineffectiveness of an increasing number of antibiotics necessitates the utilization of more potent antimicrobial agents for combatting uncomplicated infections. In response to the escalating prevalence of multidrug-resistant pathogens and the imperative to curtail the demand for novel antibiotics, the Antimicrobial Stewardship Program was conceived and implemented. This initiative is characterized by a lead physician, ideally possessing expertise in infectious diseases, alongside a pharmacist serving as a secondary leader and a microbiologist with defined responsibilities to achieve several objectives. These objectives include reducing indiscriminate usage of antimicrobial agents, promoting selective antimicrobial utilization based on culture results, de-escalating therapy from broad-spectrum to targeted antimicrobial agents, and transitioning from parenteral to oral administration when feasible. These objectives are pursued through a combination of pre-prescription and post-prescription strategies. While the Antimicrobial Stewardship Program is widely established in developed nations, a pressing need exists for its more comprehensive implementation in less developed regions. This review aims to examine the strategies used in antimicrobial stewardship programs to evaluate their effectiveness in preventing the development of multidrug-resistant organisms (MDROs) based on existing research studies. Under the Antimicrobial Stewardship Program, education of healthcare professionals and continuous disposal of information about antimicrobial resistance have helped to restrict the emergence of multidrug-resistant organisms.

## Introduction and background

Antimicrobial resistance (AMR) has become a serious global issue, posing a significant threat to public health and healthcare professionals. Since the advent of penicillin, many antibiotics have lost their effectiveness in combating microbes simply due to inappropriate, irrational, unnecessary, and unrestricted use. The first World Health Organization (WHO) global report on AMR surveillance, published in April 2014, first collected data from national and international surveillance networks and highlighted the extent of this phenomenon in many regions of the world and the great gap in existing surveillance. The WHO Global Antimicrobial Resistance and Use Surveillance System (GLASS) was launched in 2015 to foster AMR surveillance and inform strategies to contain AMR [1]. The damage caused is mortality and morbidity about the emergence of multidrug-resistant organisms (MDROs). As existing antimicrobials have become ineffective, more potent and expensive antimicrobials are needed to treat common infections at considerable cost to patients [2]. Emphasizing the importance of regulating and promoting the prudent use of antimicrobials has been imperative in response to the decreasing effectiveness of antimicrobials and the rise of MDROs [3]. For this, an Antimicrobial Stewardship Program was started with an infectious disease physician as the leader and a pharmacist as the second leader in each institution. The team is responsible for ensuring that antimicrobials are controlled to prevent the emergence of multidrug resistance without affecting the recovery of patients [4]. This review aims to examine the strategies used in antimicrobial stewardship programs to evaluate their effectiveness in preventing the development of MDROs based on existing research studies.

## Review

Antibiotic stewardship programs

Antimicrobial stewardship is a coordinated program designed to encourage the appropriate use of antimicrobials, including antibiotics. Its primary goals are to improve patient outcomes, reduce microbial resistance, and mitigate the spread of infections caused by multidrug-resistant organisms [5]. Working with healthcare professionals to observe the 5 “D”: 1) correct drug, 2) accurate dose, 3) appropriate drug route, 4) appropriate duration and timely, 5) de-escalation to antimicrobial therapy. To prevent patients from misusing antimicrobials excessively or abusively. Reduce adverse antibiotic effects. To minimize resistance. Lowering of healthcare-associated costs [5]. Taking into account the objectives mentioned above, antimicrobial stewardship programs incorporate the following essential elements (Table 1).

**Table 1:** Principles and elements of antibiotic stewardship programs [5-9]. IT: information technology

Principles and Elements	Description
Commitment to Leadership	Support from the leadership is critical for successful Antimicrobial Stewardship Program implementation. They can take various forms, such as Supportive training, education of the staff, and making finance, IT resources, and time available for the Antimicrobial stewardship program.
Accountability and Drug Expertise	The stewardship program benefits from physicians trained in infectious disease and antibiotic stewardship and designating a single pharmacist leader as co-leader to enhance outcomes.
Action	The implementation of policies that advocate for the optimal use of antibiotics, the application of specific interventions to enhance antibiotic utilization, and the prioritization of interventions based on the requirements of the healthcare setting. Adoption of policies helps to modify or discontinue antimicrobials promptly.
Tracking and Reporting of Antimicrobial Use and Outcomes	Discover improvement areas and monitor the results of improvement actions, for example, evaluating whether the prescriber correctly prescribed an antimicrobial drug for a specific indication, including a duration record and appropriate testing before treatment.
Education	Prescribers are encouraged to optimize antimicrobial prescriptions by receiving new information on antibiotic prescribing, resistance, and management of infectious diseases. Staff groups can be educated through informative presentations and electronic communications, among other methods.

Antibiotic stewardship programs in different healthcare settings

Infectious Diseases Physicians

Dedicated infectious disease-trained physician who spends most of the time designing and implementing programs. An infectious disease physician must review therapeutic recommendations, antibiotic limitation policies, and other measures to ensure that they do not pose a danger to patients. These measures are crucial in preventing the spread of antibiotic resistance and ensuring optimal patient care. Additionally, the infectious disease physician collaborates with other healthcare professionals to provide education and training on infection control practices. By staying updated on the latest research and guidelines, they can effectively contribute to the prevention and management of infectious diseases in healthcare settings [10].

Clinical and Hospital Pharmacists

Pharmacists are essential because of their role in dispensing and knowledge of the available medicines. When restricted antimicrobials are prescribed, pharmacists might notice and tell the physician that authorization is necessary. The most common source of AMR is the inappropriate use of antibiotics, which has been associated with a tendency for self-medication and unnecessary use of antibiotics for viral disease. This misuse of antibiotics can lead to the development of drug-resistant bacteria, making it difficult to treat infections effectively. Pharmacists, with their expertise in medications, play a crucial role in educating patients about the appropriate use of antibiotics and promoting responsible antibiotic stewardship. By providing information on the potential risks and benefits of these drugs, pharmacists can help prevent the spread of AMR and ensure that patients receive the right treatment for their specific condition [11].

Clinical Microbiologists

Antimicrobial stewardship programs depend largely on clinical microbiology laboratories. AMR rate data enable the antimicrobial stewardship team to assess the hospital’s existing burden of antimicrobial resistance, facilitating informed decisions regarding the restriction or evaluation of specific antimicrobial agents. By analyzing the antimicrobial resistance rate data, the antimicrobial stewardship team can identify patterns and trends in resistance, allowing them to develop strategies to combat the problem effectively. Furthermore, clinical microbiology laboratories play a crucial role in providing timely and accurate susceptibility testing, helping clinicians choose the most appropriate antimicrobial therapy for individual patients. Without the collaboration between antimicrobial stewardship programs and clinical microbiology laboratories, efforts to combat antimicrobial resistance would be significantly obstructed [4].

Staff for Infection Control and Hospital Epidemiologists

Hospital staff engaged in antimicrobial management are crucial in the global fight against AMR. Their role is to optimize the use of antibiotics and other antimicrobials within healthcare settings, preserving their effectiveness for the future. These experts develop and implement programs that encourage responsible antimicrobial use [8]. They collaborate with healthcare providers to ensure antibiotics are prescribed and administered properly, reducing the risks of overuse and misuse. They regularly assess prescribing patterns, monitor resistance trends, and offer feedback to healthcare professionals, promoting a culture of prudent antibiotic use [11].

Furthermore, they provide education and training to medical teams and raise awareness about AMR and the importance of antimicrobial management among both staff and patients. They establish guidelines and protocols for antibiotic therapy, helping clinicians make evidence-based decisions in infection treatment. Hospital staff engaged in antimicrobial management are pivotal in preventing drug-resistant pathogens, which can lead to treatment failures and increased healthcare costs. Their efforts contribute to patient safety and public health by maintaining the effectiveness of existing antimicrobial agents and reducing the spread of resistant infections. In a world where AMR is an escalating threat, their work is vital to ensure antibiotics remain effective for future generations [12].

Hospital Administrators

Institutional policy, hospital leadership approval, program finance, and physician autonomy are essential for the successful adoption of antimicrobial stewardship. The facility’s administrator should take measures such as giving enough time for individuals to manage and implement it on a daily basis and having regular meetings to review the resources required to fulfill the hospital’s goals of improving antimicrobial usage. The dedicated team consists of a physician in charge with antimicrobial use competence, a dedicated chemist with antimicrobial use knowledge, and the personnel required for other medical facilities to operate [13].

Reducing antimicrobial resistance through antibiotic stewardship programs

Reduced antibiotic resistance can be achieved through judicious use of antibiotics guided by the principles established in the Antimicrobial Stewardship Program, as well as informed by data such as antibiotic pharmacokinetics and pharmacodynamics, diagnostic assessments, antimicrobial susceptibility profiles, clinical responses and considerations of their impact on the microbiota along with advancement in new antibiotic research and development [14]. Regulating antibiotic use in food animals is critical to prevent antibiotic resistance. Strategies to manage resistance include educating the general public and healthcare professionals about the distinct characteristics of bacterial infections, promoting responsible antibiotic prescription practices, and emphasizing personal hygiene such as hand washing [15].

Decreasing Antibiotic Use

Reduced antibiotic consumption leads to reduced resistance [16]. The classic Finnish study on macrolide-resistant Streptococcus pyogenes demonstrated how reducing macrolide use could minimize antimicrobial resistance. Resistance to antibiotics fell from 9.2% in 1997 to 7.4% in 2000 [17]. The 2020 threat estimates for antimicrobial-resistant bacteria and fungi in the USA show a significant increase in hospital-onset cases, particularly for carbapenem-resistant Acinetobacter and drug-resistant Neisseria gonorrhoeae. Antifungal-resistant Candida auris also showed a significant increase. The report emphasizes the need for targeted interventions and public health measures to address the growing threat of antimicrobial resistance, compounded by COVID-19 [18]. Likewise, the analysis revealed a 41% reduction in vancomycin-resistant Enterococci, a 33% decline in Acinetobacter resistant to carbapenem, a 29% decrease in multidrug-resistant Pseudomonas aeruginosa, a 25% decrease in Candida, and a 21% decrease in methicillin-resistant Staphylococcus aureus due to antibiotic stewardship. The objective is to encourage the judicious and appropriate use of antibiotics. It also promotes sensible antibiotic use by prescribing antibiotics only to patients expected to benefit from treatment [19].

Prescription Guidelines

There are two significant approaches to prescribing recommendations for antimicrobial stewardship, with the combination of both being the most successful. The front-end or pre-prescription approach to management involves the utilization of restricted prescriptive authority [20]. Specific antimicrobials have limitations on their use and require pre-approval, with the exception of a select group of healthcare professionals [21]. This approach has led to considerable cost savings for the specific targeted medication but has also led to the use of unrestricted antimicrobials [22]. The back-end or post-prescription approach to management employs prospective review and feedback [20]. The antimicrobial steward assesses current antibiotic prescriptions and recommends whether the physician should maintain, modify, or replace the medication, considering the available microbiological data and clinical factors in each case [23]. These initiatives have shown reduced antimicrobial utilization, decreased new antimicrobial prescriptions, and increased physician satisfaction. This approach has the advantage of focusing on de-escalation, an essential feature of antimicrobial usage [24].

Restricted Antibiotic Formularies

The antimicrobial formulary list should include the antimicrobials suggested in the latest “Model List of Essential Medicines’ or the national essential medicine list, if available, with local or regional adaptation based on local infection patterns, resistance profiles of common organisms, and drug availability and affordability. A formulary is necessary within a hospital to ensure an adequate supply of essential medicines to treat the range of diseases seen within the patient population and satisfy the healthcare needs of the people [25]. All restricted antimicrobials prescribed and administered in the hospital must be closely monitored, evaluated, and reported [26]. This ensures that only authorized prescribing staff members have access to these drugs and that these medicines are being used appropriately [27]. To further assist in the development of tools for antimicrobial stewardship programs at local, national, and global levels, the Expert Committee also classified antibiotics into three groups to ensure improved access and clinical outcomes, reduce the risk of antimicrobial resistance, and preserve the use of the “last-resort” antibiotics for those who require them. These were the “Access”, “Watch”, and “Reserve” antibacterial groups [28]. The Access group is first- or second-line empiric therapy for many common indications. These are generally narrower-spectrum with a low risk of toxicity. These should be readily available in all hospitals [29]. The Watch group is thought to have a greater risk of toxicity or a higher potential to induce resistance. Antimicrobial stewardship programs should limit their use to only recommended indications [30]. In the management of severe or life-threatening infections caused by multidrug-resistant bacteria, the reserved category of drugs is utilized as a last-resort option. These drugs are to be protected against inappropriate use through strict restriction and approval programs [31]. This classification was introduced by WHO in 2017 and is updated every 2 years. WHO has updated its classification in 2023 which includes a country-level target of at least 60% of total antibiotic consumption being Access group antibiotics [32].

De-escalation Strategies

De-escalation refers to modifying the initial empirical antimicrobial regimen in response to culture data, laboratory analysis, and patients’ clinical conditions [33]. Adjustment may involve switching from a broad-spectrum antibiotic to one with a narrow spectrum, switching from combination therapy to monotherapy, or discontinuing antibiotic treatment when it is unnecessary. De-escalation was done to minimize the emergence of antibiotic resistance [34].

Direct Evidence

In patients with septic shock or severe sepsis, every additional day of exposure to piperacillin-tazobactam, meropenem, and cefepime was related to more remarkable resistance development [35]. The research included patients with ventilator-associated pneumonia in the ICU, with de-escalation occurring in 38% of cases. On day 21, the prevalence of multidrug-resistant bacteria was 14.3% with de-escalation and 21.3% with continued therapy. The researchers used propensity scoring to compare matched groups of patients [35]. They found that the incidence of antibiotic resistance during admission to the ICU was 31% for those undergoing the de-escalation, and 40.5% for those receiving continuous therapy [36].

Indirect Evidence

The gastrointestinal microbiota is a separate organ from which multidrug-resistant bacteria can emerge. The human gut microbiota acts as a medium for bacteria to share genetic material horizontally [37]. Consequently, the spread of genetic material that confers antimicrobial resistance to nonpathogenic bacteria can be just as detrimental as its presence in pathogenic bacteria, regardless of the specific bacterial hosts containing these antimicrobial resistance genes. Next-generation sequencing (NGS), polymerase chain reaction (PCR), DNA microarray, whole-genome sequencing and metagenomics, and matrix-assisted laser desorption ionization-time of flight mass spectrometry detect their presence [38]. Although imipenem is a broad-spectrum antibiotic, Woerther et al. found little effect on microbiota diversity and its resistance to colonization resistance [39]. Other antibiotics, such as piperacillin/tazobactam and ceftriaxone, have been linked to more negative effects on the gut microbiota.

Improving Antibiotics

Multidrug-resistant bacteria currently kill about 25,000 people in Europe annually, costing the economy about €1.5 billion [1]. The situation is similar in the United States, where multidrug-resistant bacteria kill nearly 23,000 of the 2 million people infected each year [40]. As the efficacy of antibiotic therapy decreases, it becomes even more essential to maintain its effectiveness and strengthen our current arsenal of antibacterial drugs. Additionally, antibiotic compliance must be improved. As a result, changes can be made by prolonging the life of current antibiotics [41]. It has already been demonstrated that drug combinations successfully combat multidrug-resistant bacteria and that antibiotic adjuvants may help protect existing drugs. The combination of amoxicillin and clavulanic acid is the most well-known and successful example [42].

Diagnostic Advancements

Early access to information on bacterial pathogens and their susceptibility promotes targeted antimicrobial therapy and shortens the duration of treatment duration [29]. Using traditional methods, the identification and susceptibility tests of bacterial pathogens usually take at least 48 hours. Delay in diagnostic procedures results in prolonged use of unsuitable and empirical antibacterial therapies. Pathogens can be identified quickly and precisely using new techniques such as susceptibility testing, DNA amplification assays, and technologies such as matrix-assisted laser desorption ionization time-of-flight mass spectrometry (MALDI-TOF), antimicrobial susceptibility testing, and Pheno test BC. Significantly, these techniques improve diagnostic accuracy while reducing the time to result, which is clearly crucial for antimicrobial stewardship [43]. The advantages of phenotypic antimicrobial susceptibility compared to genotypic testing are i) drug resistance and drug susceptibility both can be predicted and ii) it allows the quantification of a bacterial isolate’s susceptibility [44].

Enhancing Prescribing Practices

Before implementing the antibiotic stewardship program, antibiotic utilization was characterized by extended courses of intravenous treatment (IV) with minimal instances of transition to oral formulations [45]. The rate of switching to oral forms was notably low and oral administration was considered impractical in various situations. The switch to practice was prompted by antimicrobial management, which guided the optimal oral form for the IV drug and the patient’s clinical state [45]. Another important result of improved antimicrobial prescription practices was a significant 40% reduction in the overall use of restricted antimicrobial agents by the year 2020, as compared to the levels seen in 2015 [41].

Feedback and Audit

The Infectious Disease Society of America advocates for prospective audit and feedback as one of the fundamental approaches crucial for the effectiveness of antimicrobial stewardship programs. A typical prospective audit and feedback program requires the participation of a multidisciplinary team, which typically includes a physician, often specializing in infectious diseases, a microbiologist, and a clinical pharmacist [46]. In general, an antimicrobial stewardship team member attends clinical rounds and gives direct feedback, often focusing on high-end antibiotics, in a one-step prospective audit and feedback [25]. An antimicrobial stewardship team member will independently analyze cases from a specific ward or critical care unit in the more thorough two-step procedure. Step 1: establish the precise goals of the Antimicrobial Stewardship Program before you begin and Step 2: prior to beginning, select metrics, evaluate performance to date, and identify any obstacles to the prudent and reasonable use of antibiotics. Following that, cases meeting the intervention criteria will be submitted to a senior team member, who will convey to the treating physician the antimicrobial stewardship team’s recommendations for treatment adjustment or antibiotic withdrawal [4].

Infection Control Measures

Hygiene and barrier precautions is a comprehensive study by Wilson et al. of the efficacy of domestic and industrial laundering of healthcare worker uniforms, which harbor infectious organisms, revealed that domestic laundering decreases microbial contamination by up to 109 [47]. Hardy et al. investigated the relationship between environmental contamination and patient infection with methicillin-resistant S. aureus and discovered a positive correlation between infection rate and environmental contamination. During each environmental screening, methicillin-resistant S. aureus was isolated, and in most cases, at least one patient in the ICU was positive [48]. The retrospective study by Huang et al. in eight different ICUs over two years showed that occupants who harbored resistant organisms contaminated inanimate objects in the room. The person occupying that room afterward gets infected by drug-resistant organisms [49].

Evidence of Antimicrobial Stewardship Program effectiveness

Improved Patient Outcomes

The most significant outcome of the Antimicrobial Stewardship Program is the decrease in the rate of hospital-acquired infections caused by multidrug-resistant organisms, and this achievement was particularly notable in the intensive care unit (ICU), where there was a remarkable 65% reduction observed by 2020 compared to 2015 [50]. Consequently, the marked reduction in the multidrug-resistant rate can be attributed to the quantitative and qualitative optimization of antimicrobial prescription and utilization facilitated by the Antimicrobial Stewardship Program [50].

Economic Benefits

Despite increased consumption in 2016, implementing the Antimicrobial Stewardship Program resulted in an early marked decrease in antimicrobial costs [51]. This is most likely a result of the Antimicrobial Stewardship Team’s efforts to improve the quality of antimicrobial prescribing. By 2020, adjusted costs per patient day had fallen by more than 50%, for a total cost reduction of US$1.50 million based on fixed medication prices, reflecting an annual average of US$300,000. A comprehensive analysis by Alawi et al., which comprised 146 research studies from all continents, found that the Antimicrobial Stewardship Program resulted in a substantial reduction in antimicrobial spending in 92% of the trials, with reductions in costs reaching up to 80% [51].

Antimicrobial Stewardship Programs and one health approach

Antimicrobial resistance is a global public health issue that has resulted in the establishment of epidemiological surveillance systems. The WHO created the GLASS in 2015 to cover knowledge gaps and guide initiatives at all levels [52]. GLASS was created with the objective of progressively integrating surveillance data concerning the use of antimicrobial agents in humans. Its purpose is to monitor patterns in antimicrobial consumption and investigate the impact of antimicrobial resistance within the food supply chain and the environment. It offers a standardized approach for collecting, analyzing, and interpreting national, regional, and local data. This allows for monitoring the advancements in both new and existing national surveillance systems [21]. In 2017, the WHO released a list of priority diseases with the aim of directing research and development of novel antibiotics, diagnostic tools, and vaccines [53].

Challenges and barriers

Implementation Hurdles

The launch of initiatives in the medical field faces significant challenges due to intense competition between doctors, driven mainly by the fear of losing patients. However, if doctors commit themselves collectively to avoiding unnecessary use of antimicrobials, the antimicrobial management program can be stimulated [23]. An antibiogram is an overall profile of antimicrobial susceptibility testing results of a specific microorganism to a battery of antimicrobial drugs. Antibiogram Utility Value in which the pharmacist believes obtaining an antibiogram, representing the community’s resistance pattern, is difficult. They attribute it to the empirical antibiotic treatment without sending for culture sensitivity [54]. Poor Regulation Enforcement focusing solely on hospitals with a policy or program may be ineffective, as stated by the pharmacologist: “When patients are denied antibiotics in hospital settings, they may prompt return to a pharmacy, where pharmacists may provide them with requested medications” [55]. Time restrictions pre-authorization is one example of a stewardship step that doctors believe may be difficult to apply, especially in a crowded outpatient department [56]. Minimal facilities for support hospital pharmacies, both primary and secondary, are usually not computerized. Because it must be done manually, quantifying and documenting antibiotic usage is frequently difficult [23].

Resource Constraints

Low- and middle-income countries face substantial hurdles, such as limited healthcare infrastructure, and high patient-to-provider ratios [57]. These are the same countries that bear the burden of high antimicrobial consumption and, as a result, high antimicrobial resistance rates. Although antimicrobial stewardship programs are desperately needed in these countries, they are frequently inadequate. According to a 2013 survey conducted by the Indian Council of Medical Research, it was revealed that among 20 tertiary-level healthcare institutes, only 40% had established written Antimicrobial Stewardship Program documents, 75% had infection control guidelines, 65% had prescription guidelines, and a mere 30% had implementation strategies in place [58]. An international African survey found that only 14% of hospitals ran antimicrobial stewardship programs. Factors like overcrowding, incomplete implementation of infection prevention measures, absence of electronic medical record systems, and a shortage of dedicated staff are often overlooked but constitute crucial barriers to effective antimicrobial stewardship initiatives [59]. Microbiology laboratories that meet the criteria for infrastructure, well-trained and experienced personnel, and quality control systems are frequently scarce, especially in rural regions [60].

## Conclusions

As more and more organisms started developing resistance to antimicrobial agents, the need for a standard approach to antimicrobial prescribing was felt, giving rise to an Antimicrobial Stewardship Program. Under the Antimicrobial Stewardship Program, education of healthcare professionals and continuous disposal of information about antimicrobial resistance have helped to restrict the emergence of multidrug-resistant organisms. Antimicrobial stewardship program using a combined front-end or pre-prescription approach and back-end or post-prescription approach has reduced the incidence of higher broad-spectrum antimicrobials for a longer duration, saving cost to the patient. De-escalation in the form of a broad spectrum of specific antimicrobials based on culture and from parental to oral administration is showing the expected results.
